# Accuracy of popular automatic QT Interval algorithms assessed by a 'Gold Standard' and comparison with a Novel method: computer simulation study

**DOI:** 10.1186/1471-2261-5-29

**Published:** 2005-09-26

**Authors:** Anthony Charles Hunt

**Affiliations:** 1 PSI HeartSignals Ltd, Institute of Medical Technology, Glasgow Technology Park, PO Box 7043, Glasgow G44 9AB. UK

## Abstract

**Background:**

Accurate measurement of the QT interval is very important from a clinical and pharmaceutical drug safety screening perspective. Expert manual measurement is both imprecise and imperfectly reproducible, yet it is used as the reference standard to assess the accuracy of current automatic computer algorithms, which thus produce reproducible but incorrect measurements of the QT interval. There is a scientific imperative to evaluate the most commonly used algorithms with an accurate and objective 'gold standard' and investigate novel automatic algorithms if the commonly used algorithms are found to be deficient.

**Methods:**

This study uses a validated computer simulation of 8 different noise contaminated ECG waveforms (with known QT intervals of 461 and 495 ms), generated from a cell array using Luo-Rudy membrane kinetics and the Crank-Nicholson method, as a reference standard to assess the accuracy of commonly used QT measurement algorithms. Each ECG contaminated with 39 mixtures of noise at 3 levels of intensity was first filtered then subjected to three threshold methods (T1, T2, T3), two T wave slope methods (S1, S2) and a Novel method. The reproducibility and accuracy of each algorithm was compared for each ECG.

**Results:**

The coefficient of variation for methods T1, T2, T3, S1, S2 and Novel were 0.36, 0.23, 1.9, 0.93, 0.92 and 0.62 respectively. For ECGs of real QT interval 461 ms the methods T1, T2, T3, S1, S2 and Novel calculated the mean QT intervals(standard deviations) to be 379.4(1.29), 368.5(0.8), 401.3(8.4), 358.9(4.8), 381.5(4.6) and 464(4.9) ms respectively. For ECGs of real QT interval 495 ms the methods T1, T2, T3, S1, S2 and Novel calculated the mean QT intervals(standard deviations) to be 396.9(1.7), 387.2(0.97), 424.9(8.7), 386.7(2.2), 396.8(2.8) and 493(0.97) ms respectively. These results showed significant differences between means at >95% confidence level. Shifting ECG baselines caused large errors of QT interval with T1 and T2 but no error with Novel.

**Conclusion:**

The algorithms T2, T1 and Novel gave low coefficients of variation for QT measurement. The Novel technique gave the most accurate measurement of QT interval, T3 (a differential threshold method) was the next most accurate by a large margin. The objective and accurate 'gold standard' presented in this paper may be useful to assess new QT measurement algorithms. The Novel algorithm may prove to be more accurate and reliable method to measure the QT interval.

## Background

The QT interval is measured as the time interval between the onset of the QRS complex and the end of the T wave, the end of the T wave being the time at which repolarisation is completed and the T wave voltage amplitude returns to the baseline [1]. The QT interval is thus a measure of the duration of the ventricular depolarisation and repolarisation. Inaccuracies occur in the measurement of the QT interval due to the low frequency content of the T wave offset, which has a low signal to noise ratio. Also the presence of a U wave merging with the end of the T wave lead to inaccuracies [2].

Accurate measurement of the QT interval is very important from clinical and pharmaceutical drug safety screening perspective, as prolongation of repolarisation, manifested by prolongation of this interval, increases susceptibility to potentially fatal torsade de pointes ventricular arrhythmia [3,4]. A statistically significant increase in the mean QT interval (corrected for heart rate) as small as 6 milliseconds between baseline and maximal drug effect may be important as a signal of repolarisation abnormality [5]. Expert manual measurement of this interval is both imprecise and poorly reproducible, inter-operator differences of up to 28 milliseconds have been reported [6]. Automatic QT interval measurements have been shown to be more stable and reproducible than manual measurement [7]. It is not surprising that manual assessment of QT dispersion has also been shown to have very poor reproducibility [8], QT dispersion being derived from the difference between maximum and minimum QT interval in a 12 lead ECG. Yet manual measurement continues to be used as a meter to assess the accuracy of much needed reliable automatic computer algorithms to measure this interval reproducibly. For a given ECG, the very fact that the manual QT interval measurement presents a varying, dynamic reference standard means that manual measurements are imprecise and non-reproducible. The degree of manual measurement imprecision is currently unknown since there has not been an accurate reproducible reference standard with which to make an assessment.

Currently available automatic computer algorithms which measure the QT interval on a given ECG will have a reasonably good reproducibility but the QT interval measured may be reproducibily incorrect. Earlier efforts by Willems et al to evaluate the performance of ECG computer measurement programs and provide a common standard were based on the stability of results or reproducibilty [9]. In the report by Willems et al, the median result of measurements made by 5 Cardiologist referees was used to assess the median results of combined computer programme measurements. The referees determined the end of the T wave a mean 5–15 ms later than the combined computer algorithms but because a subjective manual reference system was used it is unknown as to whether the combined referee of combined computer programs was more accurate in determining the real T wave offset.

Presented in this research is an objective reference standard against which the accuracy and reproducibility of commonly used QT measurement algorithms can be assessed. This suggested new 'gold standard' uses a validated computer simulated electrocardiographic waveform [10] generated from a cell matrix using validated Luo-Rudy membrane kinetics [11]. The simulated ECG allows the exact timing of the cessation of repolarisation or end-point of the T wave to the nearest 0.005 milliseconds. ECGs will be constructed to simulate the varying intramyocardial conductivity and myocardial volume effects, in addition to extramyocardial conductance effects. Superadded to the simulated ECGs will be mixtures of three simulated different types of noise at different intensities [12,13]. Six different algorithms will be used to measure T wave offset [14], including a Novel algorithm. All algorithms will be statistically compared for reproducibility and accuracy to determine the underlying real QT interval for each ECG.

## Methods

Initially four ECGs were constructed to simulate lumped variations in the electrophysiological properties of myocardial type and myocardial fibre orientation. The four ECGs constructed represented all the permutations of a high myocardial resistivity (100,000 Ohm cm), low resistivity (10,000 Ohm cm), time-dependent potassium channel conductance of 584 mS/cm^2 ^and time dependent potassium channel conductance 262 mS/cm^2^. Variations in the ratio of density of the rapid and delayed potassium rectifier channels exist within different strata of the myocardium and a change in the ratio of these channels will therefore produce a different total conductance [15]. Variation in fibre orientation will produce a variation in resistivity.

Each of the four models of myocardial fibres with combinations of different resistivities and conductances consisted of 100 individually calculated cardiac cell membrane potentials, interconnected via resistors which represent gap junctions. For each myocardial fibre model the membrane ion kinetics were computed at an extracellular potassium concentration of 5.4 mmols using the meticulous Luo-Rudy physiological model which has been validated by experimental data [11]. The current propagation in one direction along each of the homogenous myocardial fibre types was evaluated by solving numerically the parabolic partial differential equation shown below (equation 1) for the transmembrane potential Vm, using the Crank-Nicholson implicit method [16], as was used in the insightful computer model by Virag et al [10]. The Crank-Nicholson implicit method has the advantage of stability irrespective of the time increments used in the iterations and provides improved accuracy at the expense of requiring the solution of a set of simultaneous equations at each time step.

Equation 1.



This equations describes current flow only intracellularly and the extracellular current is grounded [17] Cm is the membrane capacitance (1 μF/cm^2^), Sv is the membrane surface area to intracellular volume ratio (0.24 μm^-1^), Iion is the sum of the fast sodium, slow inward calcium, time dependent potassium, time independent potassium, plateau potassium and background membrane ionic currents in (μAmps/cm^2^), Istim is the stimulus current in (μAmps/cm^2^) and px is the resistivity in the x direction (10,000 and 100,000 Ohm cm). Equation 1 is discretised to equation 2 shown below.

Equation 2.

Vm^i,t+Δt ^- Vm^i,t ^+ (Iion^i,t ^- Istim^i,t^)Δt/Cm^i ^= r/2(Vm^i-1,t+Δt ^- 2 Vm^i,t+Δt ^+ Vm^i+1,t+Δt ^+ Vm^i-1,t ^- 2 Vm^i,t ^+ Vm^i+1,t^)

In equation 2, Δt is the iteration time step of 0.005 milliseconds and Δx is the space discretisation of the grid (100 μm). The partial derivative of Vm being written as the finite difference (Vm^i,t+Δt ^- Vm^i,t^)/Δt.

The symbol r is the coupling factor = Δt/Cm^i ^Sv (Δx)^2^px. The superscript i is the cell position in a grid of 100 cells. The superscript t is the time elapsed from onset of the simulation, Δt is the iteration time increment of 0.005 milliseconds.

The computational method is iterative in time following two steps, firstly the total ionic current is computed for each cell for each Vm^i,t ^using the Luo- Rudy membrane kinetics. Calculation of the ionic current for each cell for each iteration is a major time limiting step, this process was accelerated at the time Vm^i,t+Δt ^reached each individual cell threshold potential of greater than -60 mV, by the use of a pre-calculated action potential (customised to the electrophysiological parameters) lookup table which gives the ionic currents for each corresponding voltage at each 0.005 millisecond time step. The set of simultaneous equations produced by each iteration of Equation 2 of for each cell was solved by the implicit method using tridiagonal matrices. Von Neumann boundary conditions are adopted which means no current flows out of either end of the cell array.

The ECG voltage potentials for the four electrophysiological conditions of varying resistivities and time dependent potassium current conductances was calculated by representing each pair of adjacent cell elements within the array as constituting an electric dipole of length Δx, the current density depending on the difference of potential (Vm^i,t ^- Vm^i+1,t^) between two adjacent cells and the resistivity of their gap junction. Given a constant electrode orientation along the x axis, the instantaneous potential recorded from a remote electrode is proportional to the sum of the dipole moments or dipole potential differences over the cell array distance calculated for each iteration time [18].

Each of the four different ECGs generated along a single vector can be used to simulate orthogonal XYZ vector ECGs through a volume of myocardium with inhomogeneous electrophysiological characteristics within each orthogonal plane of myocardium. By the addition of each XYZ potential voltage generated every 0.005 milliseconds for each of the XYZ vector ECGs it is possible to derive a resultant vector ECG for the volume of myocardium simulated. Using three combinations of ECGs derived under different electrophysiological conditions and positive or negative orientations it is possible to produce complex resultant ECG T waves. Two predominantly positive T waves (Res1 and Res2) and two biphasic T waves (Res3 and Res4) were simulated.

Extracellular unipolar potentials Φ generated by a myocardial fibre orientated along a vector axis x in an extensive medium of extracellular conductivity σ_e _are computed from the transmembrane potential Vm using equation 3 [19].

Equation 3 Φ = a^2 ^σ_i _(∫ (-grad(Vm))(grad(1/r))dx)/4 σ_e_

Where a is the cross sectional area of the fibre, σ_i _is the intracellular conductivity, r is the distance from the point source to the field point and grad is the grad vector operator. It can be seen that none of the variables and operators in equation 3 contain the variable of time. Therefore a change in magnitude of σ_e _or other dimensions of length for a given σ_i _and Vm will have a linear effect and directly proportional effect on the amplitude of Φ without any effect on the time duration. This has been experimentally borne out by Mirvis et al [18].

Therefore to simulate the effects of a doubling of σ_e _for the four resultant ECGs generated the ECG voltage potential was doubled every 0.005 milliseconds of the ECG complex duration. All simulated ECGs voltages below 0.00001 millivolts were reduced to zero.

### Noise simulations

Mixtures of three types of noise at three intensity levels, producing 39 different combinations of noise, were added to each of the eight simulated ECGs. Creating a total of 312 ECGs analysed. The three types of noise contaminating the ECG with their respective frequencies at signal to noise ratios (SNR) of 30, 40 and 50 dBs, comprised: Mains noise (50 Hz), electromyographic noise (Gaussian white noise) and respiratory noise (0.25 and 0.5 Hz). Tikkanen et al, using simulated noise to define optimal QT intervals of noisy ambulatory ECGs, used an SNR range of 5–50 dB. Levels of SNR in the lower half of this range were used to simulate the extremely noisy tracings of active ambulatory patients [12]. As it was intended to simulate noise on resting ECGs in this study, an SNR level starting at 30 dB more accurately simulate the noise levels found on resting ECGs.

Baseline wander due to respiration at rates of 30 and 15 breaths per minute were simulated by a sine wave of 0.5 and 0.25 Hz with lag phases of pi/2, pi and 3pi/2 radians and respiratory modulation of 15% [12]. The highest rate of respiration was the same in the Tikkanen study to simulate a mildly breathless patient.

The SNR was calculated as 20log(standard deviation of the baseline signal/standard deviation of added noise). Abrupt step effects due to motion artefacts were not modelled as resting ECGs were simulated in this study. The mains noise was simulated using a 50 Hz sine wave, harmonics of the powerline frequencies were not modelled as the 50 Hz frequency would be dominant [13]. Gaussian white noise was simulated using a random noise generator, the respiratory effects of sinusoidal baseline wander and amplitude modulation were combined and simulated from the following function: (1 + A(sine(2(pi)R + ϕ))y(t).

Where A is the modulation index at 15%, R is the respiratory frequency (15 or 30) breaths per minute, ϕ is the phase lag of pi/2, pi and 3pi/2 radians and y(t) is the function of uncontaminated simulated ECG as a function of time. The abbreviations which describe different combinations of the three noise types at various intensities are shown below.

N0 = The baseline simulated ECG without noise

N1 = Baseline + 50 dB SNR mains noise.

N2 = Baseline + 40 dB SNR mains noise.

N3 = Baseline + 30 dB SNR mains noise.

N4 = Baseline + 50 dB SNR white noise.

N5 = Baseline + 40 db SNR white noise.

N6 = Baseline + 30 db SNR white noise.

N7 = Baseline + Respiration 15/min + pi/2 phase.

N8 = Baseline + Respiration 15/min + pi phase.

N9 = Baseline + Respiration 15/min + 3pi/2 phase.

N10 = Baseline + Respiration 30/min + pi/2 phase.

N11 = Baseline + Respiration 30/min + pi phase.

N12 = Baseline + Respiration 30/min + 3pi/2 phase.

N13 = Baseline + 50 dB SNR white noise + 30 dB SNR mains noise.

N14 = Baseline + 50 dB SNR white noise + 30 dB SNR mains noise.

N15 = Baseline + 50 dB SNR white noise + 30 dB SNR mains noise.

N16 = Baseline + Respiration 30/min + pi/2 phase + 30 dB SNRwhite noise.

N17 = Baseline + Respiration 30/min + pi/2 phase + 30 dB SNR mains noise.

N18 = Baseline + Respiration 30/min + pi/2 phase + 30 dB SNR white noise +30 dB SNR mains noise.

N19 = Baseline + Respiration 30/min + pi phase + 30 dB SNR white noise.

N20 = Baseline + Respiration 30/min + pi phase + 30 dB SNR mains noise.

N21 = Baseline + Respiration 30/min + pi phase + 30 dB SNR mains noise + 30 dB SNR white noise.

N22 = Baseline + Respiration 30/min + 3pi/2 phase + 30 dB SNR white noise.

N23 = Baseline + Respiration 30/min + 3pi/2 phase + 30 dB SNR mains noise.

N24 = Baseline + Respiration 30/min + 3pi/2 phase + 30 dB SNR mains noise + 30 dB SNR white noise.

N25 = Baseline + Respiration 15/min + 0 phase + 30 dB SNR white noise.

N26 = Baseline + Respiration 15/min + 0 phase + 30 dB SNR mains noise.

N27 = Baseline + Respiration 15/min + 0 phase + 30 dB SNR mains noise + 30 dB SNR white noise.

N28 = Baseline + Respiration 15/min + pi/2 phase + 30 dB SNR white noise.

N29 = Baseline + Respiration 15/min + pi/2 phase + 30 dB SNR mains noise.

N30 = Baseline + Respiration 15/min + pi/2 phase + 30 dB SNR mains noise + 30 dB SNR white noise.

N31 = Baseline + Respiration 15/min + pi phase + 30 dB SNR white noise.

N32 = Baseline + Respiration 15/min + pi phase + 30 dB SNR mains noise.

N33 = Baseline + Respiration 15/min + pi phase + 30 dB SNR mains noise + 30 dB SNR white noise.

N34 = Baseline + Respiration 15/min + 3pi/2 phase + 30 dB SNR white noise.

N35 = Baseline + Respiration 15/min + 3pi/2 phase + 30 dB SNR mains noise.

N36 = Baseline + Respiration 15/min + 3pi/2 phase + 30 dB SNR mains noise + 30 dB SNR white noise.

N37 = Baseline + Respiration 30/min + 0 phase + 30 dB SNR white noise.

N38 = Baseline + Respiration 30/min + 0 phase + 30 dB SNR mains noise.

N39 = Baseline + Respiration 30/min + 0 phase + 30 dB SNR white noise + 30 dB SNR mains noise.

### Automatic computer algorithm

Prior to the algorithm quantifying the QT intervals, the signals N1 to N39 were smoothed using a moving median smoother over 21 consecutive time points. The residuals from the first smoothing operation were then smoothed in the same fashion as the original smoothed vector and the residual smoothed vector were added. This smoothing operation enabled low pass filtering without phase distortion. The uncontaminated T wave offset contains only low frequency signals and therefore removal of the high frequency noisy signals and absence of phase distortion would theoretically have minimal effect on the real T wave offset hidden within the noise contaminated ECG [20].

Four automatic computer algorithms were used to determine the ECG T wave offsets and have been described previously [14,20]. The threshold technique determines the end of the T wave as that time point when the ECG signal crosses the threshold at 5% the amplitude of the peak T wave (T1) or when the ECG crosses the threshold at 15% the amplitude of the peak T wave (T2). These ranges were used in the study by McLaughlin et al.

The differential threshold technique, is a method whereby the T wave end is determined as the interception of the first differential of the T wave with respect to time with the zero isoelectric line (T3). Two other commonly used algorithms based on the slope features of the T wave were also used: The slope intercept technique identifies the end of the T wave as the intercept of the line tangential to the point of maximum T wave down-slope with the isoelectric line and the least slope intercept method calculates a least squares fitted line of 8 milliseconds duration around the region of the maximum slope point and the time of intersection of this line with the zero isoelectric line is deemed the end of the T wave.

The Novel algorithm first involves a filtering process (fully described below). Following the filtering process which removes noise and produces a filtered signal the down-slope of the T wave ends when it becomes an isoelectric (that is line of zero voltage gradient ie constant millivoltage) baseline. The algorithm detects the first four milliseconds of the filtered signal which becomes a constant voltage (isoelectric) and then this four seconds of baseline plus any ensuing baseline up to the next P wave, are best least squares fit to the same duration of filtered inverted image baseline. The Novel algorithm is based on the axiomatic principle that the T wave end point is that first point of intersection (overlap) of the T wave with a superimposed inverted image of itself i.e. when both the T wave and its inverted image first coincide and return to a common baseline (the isoelectric line). This occurs when there is best least squares fit between both the T wave and its inverted image along the isoelectric line within the TP segment. This method is equivalent to using the T wave as a template which measures itself.

As is standard practice in commercial ECG machines the raw waveform was first pre-processed by a low pass filter, before any of the six algorithms were applied to the noisy ECG. This was performed by median smoothing as described previously.

The signal was then further smoothed by applying a zero-phase Butterworth 125^th ^order low pass filter with an adaptive iterative low pass algorithm which expands the low pass threshold between a range of 30–40 Hertz by increments of 0.1 Hertz. Each iteration of filtering can be described as repetitive looping of the signal through a low pass filter, the low pass range incrementing by 0.1 Hertz during each loop. After each iteration, fitting coefficients were calculated between the 30 millisecond segments (beginning at the time of maximum negative gradient for each T wave down-slope) of the pre-filtered signal and the filtered ECG waveform produced at that iteration. The filtered waveform exhibiting the best fit between the segments of maximum down-slopes of the unfiltered and filtered T waves were then thresholded by reducing all values below 0.00001 millivolts to zero, consensual with the thresholding applied to all the computer generated ECGs. The zero phase filtering was performed to smooth the ECG isoelectric baseline maximally whilst preserving the correspondence between the T waves of the pre-filtered and filtered signal as accurately as possible. Following the low pass filtering, the image of the filtered signal demonstrating best fit with the pre-filtered signal (over the 30 millisecond segment beginning at time of maximum negative T wave downslope) was inverted and this inverted image of the filtered signal was then vertically shifted as necessary towards the upright filtered signal to the position of best least squares fit between their respective TP isoelectric (that is line of zero voltage gradient ie constant millivoltage) baselines. The TP baseline is detected by the algorithm as the first four milliseconds of the filtered signal which becomes a constant voltage and then this four milliseconds of baseline plus any ensuing baseline are best least squares fit to the same duration of filtered inverted image baseline The end of the T wave was deemed as the first time point of intersection (overlap) between the two respective isoelectric lines.

**Figure 1:**
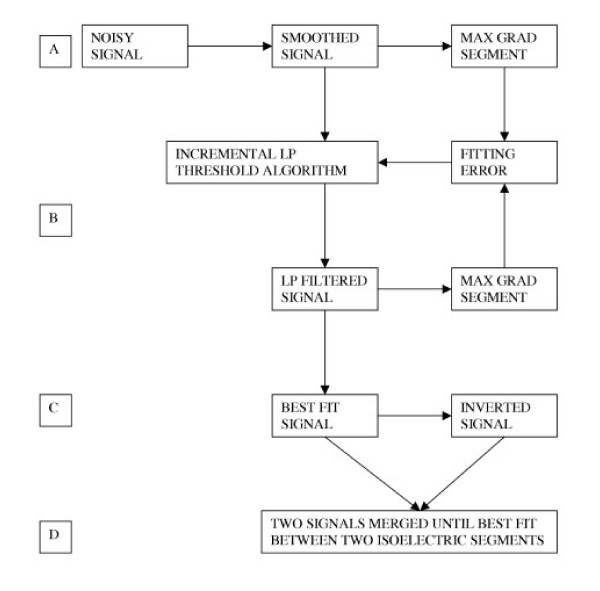
**Block diagram showing the Novel algorithm process**. The Max grad segment refers to the 30 millisecond segment starting from the time of maximum gradient measured on the pre-filtered T wave downslope. Step A describes smoothing of the raw signal and calculation of Max grad. Step B is the iteration or looping of the algorithm as described in the text. LP is low Pass filter. Step C is the generation of a filtered signal with best fit between its Max grad and that of the smoothed signal. Step D is the overlapping between the isoelectric TP baseline segments of the signal generated in C and its inverted counterpart.

**Figure 2:**
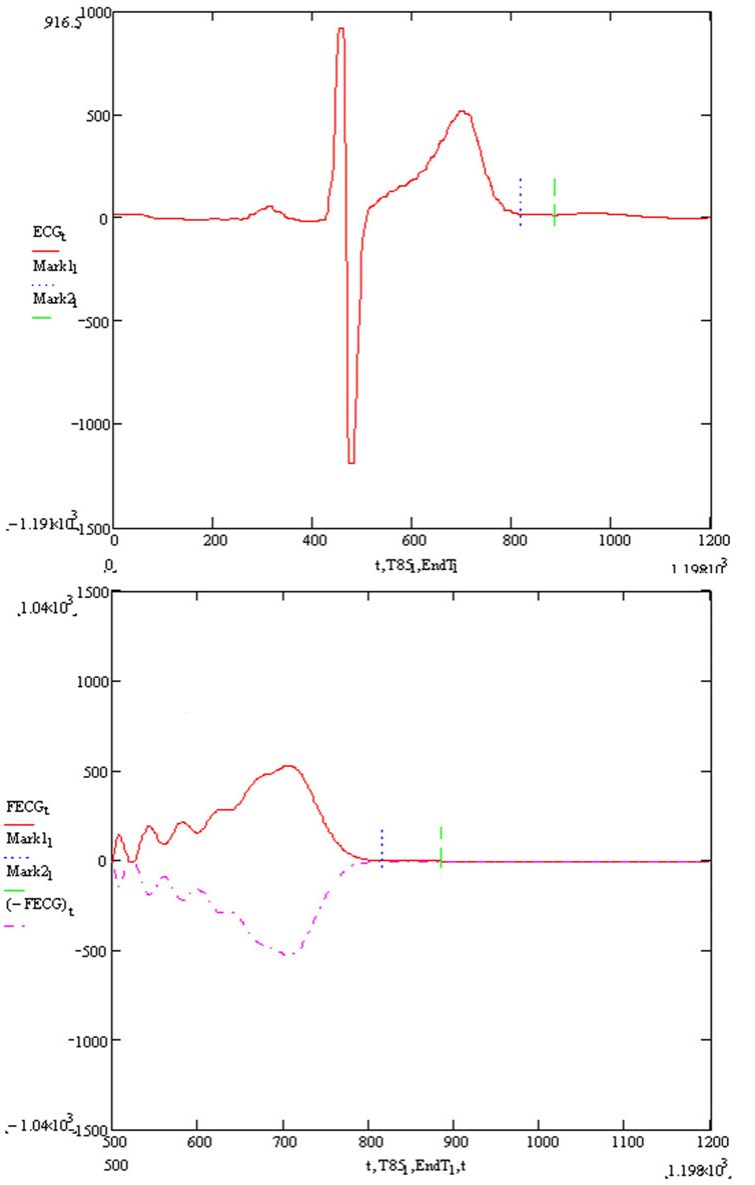
**Example of raw in-vivo signal undergoing Novel algorithm process**. The upper figure shows a raw human digitised ECG signal undergoing processing by the Novel algorithm. The first vertical line (T85) is sited at a point 85% the duration of the QT interval measured using the T wave end calculated by the Novel algorithm. The second vertical line (EndT) shows the end of the T wave as calculated by the Novel algorithm. The lower figure shows the upright and inverted T wave signals (generated in step C) undergoing step D in Figure 1. The vertical lines T85 and EndT have the same significance as in the upper part of the Figure 2.

Figure 1 is a block diagram illustrating the process of the algorithm and Figure 2 illustrates step D in Figure 1 applied to an in-vivo signal.

The biphasic simulated ECGs were first processed by the following method, prior to application of the automatic computer algorithm: Amplitudes were first squared over their sampling times and then a square root was taken. This manipulation had the effect of converting the biphasic positive/negative phases of the T wave into positive/positive biphasic T waves. The automatic computer algorithm was applied to the second positive phase of the T wave.

### Statistical analysis

The six different automatic computer algorithms were used to calculate the QT intervals to the nearest millisecond on each of the eight simulated ECGs contaminated with 39 different noise combinations. The mean, standard deviation and coefficient of variation (to compensate for the relative difference in QT interval magnitudes using different algorithms) were calculated and statistically compared. The single factor ANOVA was used to first identify any significant difference between the mean QT intervals calculated for the noise contaminated simulated ECGs using the different automatic algorithms. The Tukey test was then used to analyse the significant differences between each comparison.

Simulations were programmed using Mathcad 2001 mathematical and signal processing software package and run on an Advent Pentium 4, 3 GHertz processor. Statistical analysis was performed using AXUM 7 software.

## Results

### The simulated ECGs

**Figure 3:**
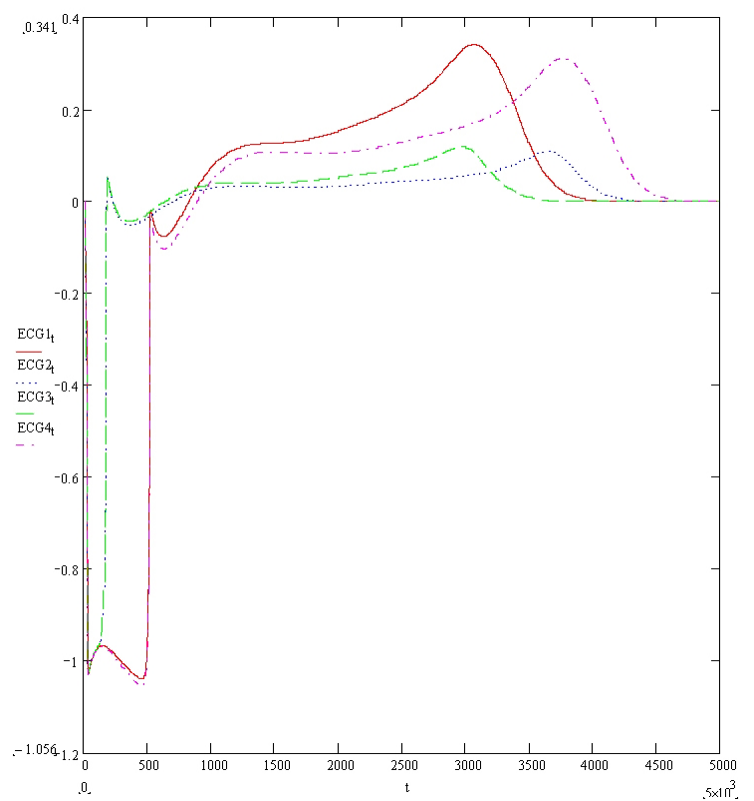
**The four simulated baseline ECGs**. ECG1, ECG2, ECG3 and ECG4 have the following (Time dependent potassium channel conductances in mS/cm^2^, Resistivities in Ohm cm): (584,100,000), (262,10,000), (584,10,000) and (262,100,000). The y axis is in millivolts and the x axis is in tenth of milliseconds.

**Figure 4:**
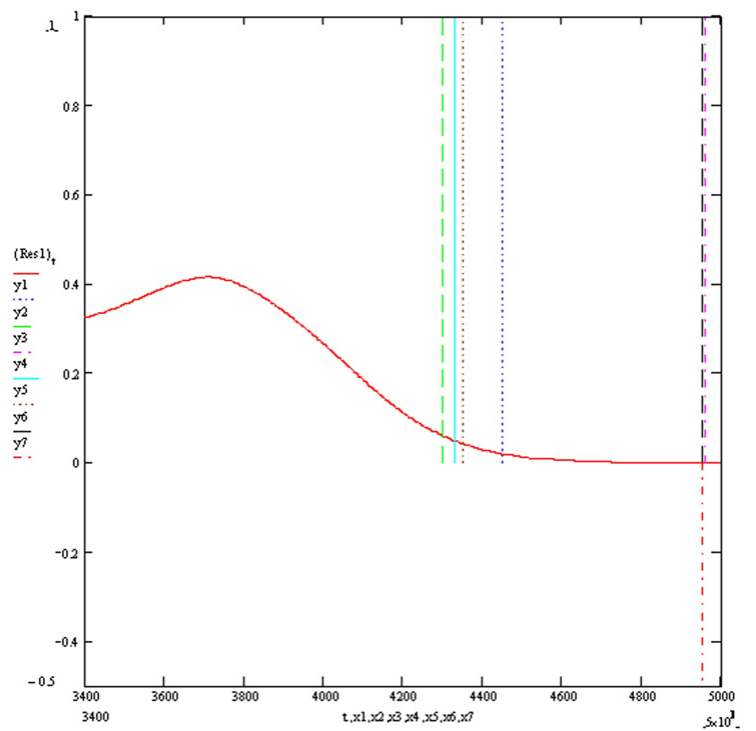
**Resultant Vector ECG Res1**. The vertical markers y1, y2, y3, y4, y5 and y6 above the electrical baseline show the calculated QT intervals by algorithms T1, T2, T3, S1, S2 and Novel respectively. The vertical marker y7 below the electrical baseline indicates the real end of the QT interval. The y axis is in millivolts and the x axis in milliseconds.

**Figure 5:**
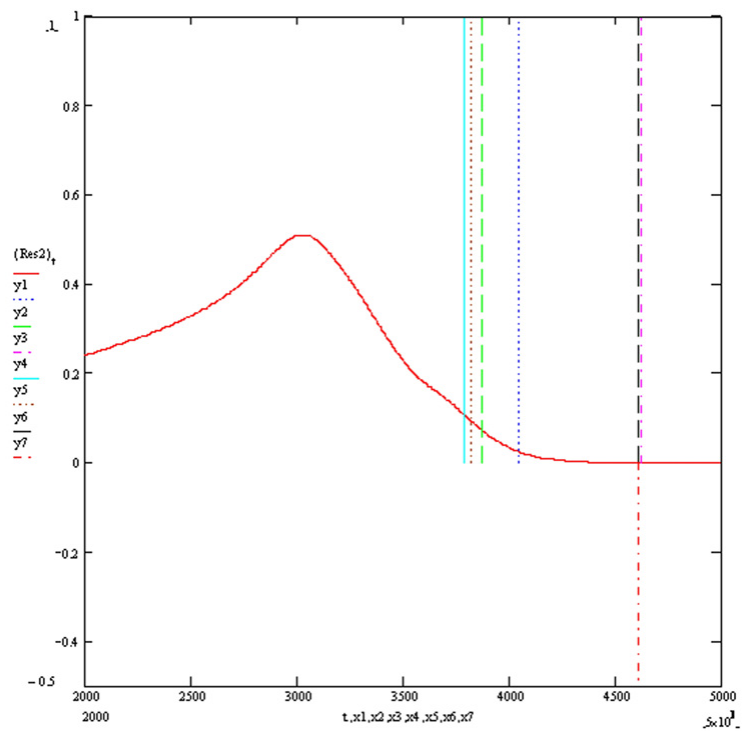
**Resultant Vector ECG Res2**. The vertical markers y1, y2, y3, y4, y5 and y6 above the electrical baseline show the calculated QT intervals by algorithms T1, T2, T3, S1, S2 and Novel respectively. The vertical marker y7 below the electrical baseline indicates the real end of the QT interval. The y axis is in millivolts and the x axis in milliseconds.

**Figure 6:**
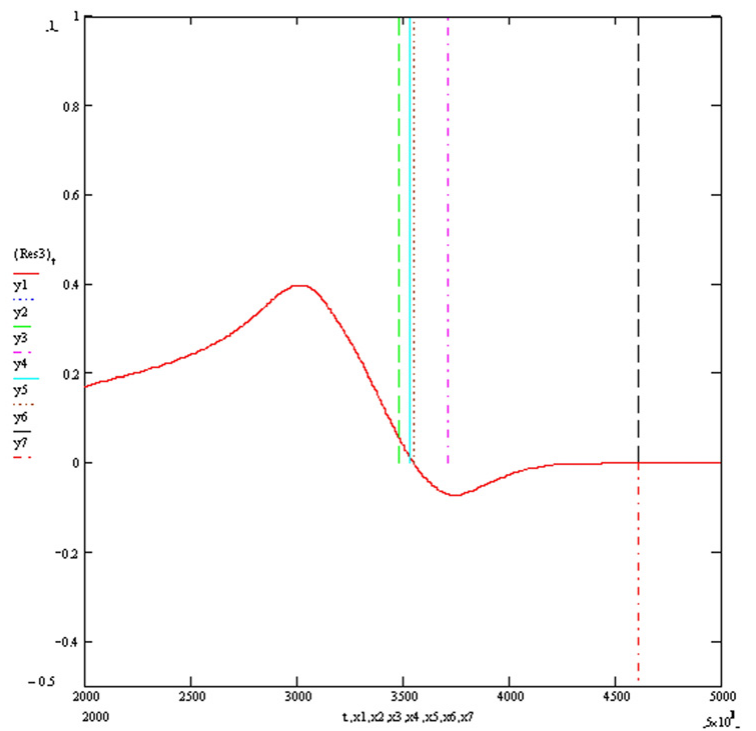
**Resultant Vector ECG Res3**. The vertical markers y1, y2, y3, y4, y5 and y6 above the electrical baseline show the calculated QT intervals by algorithms T1, T2, T3, S1, S2 and Novel respectively. The vertical marker y7 below the electrical baseline indicates the real end of the QT interval. The y axis is in millivolts and the x axis in milliseconds.

**Figure 7:**
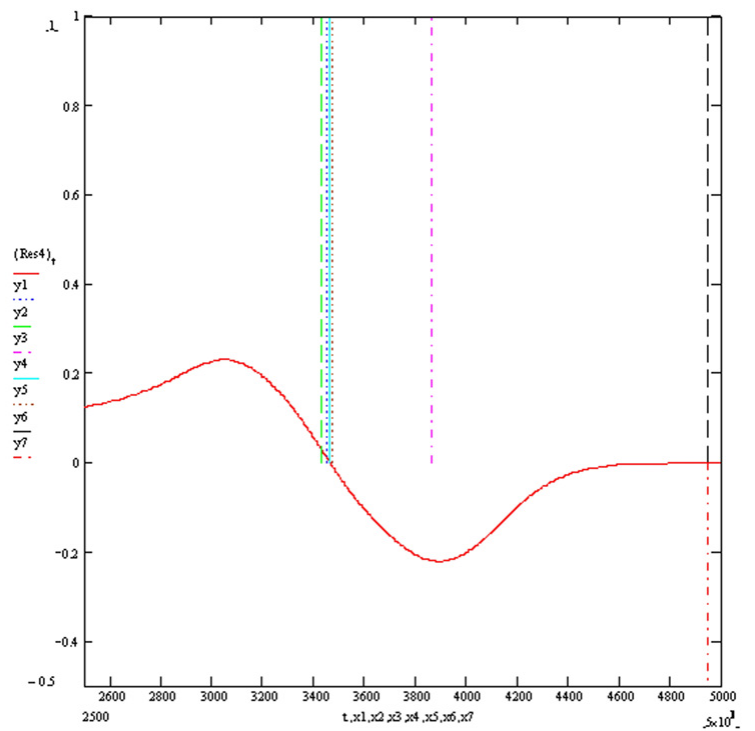
**Resultant Vector ECG Res4**. The vertical markers y1, y2, y3, y4, y5 and y6 above the electrical baseline show the calculated QT intervals by algorithms T1, T2, T3, S1, S2 and Novel respectively. The vertical marker y7 below the electrical baseline indicates the real end of the QT interval. The y axis is in millivolts and the x axis in milliseconds.

Figure 3 shows the four baseline ECGs (ECGs1, 2, 3 and 4) simulated at an extracellular potassium concentration of 5.4 mmols with different combinations of resistivities and potassium channel conductances. ECGs 1, 2, 3 and 4 were simulated with respective (time dependent potassium channel conductances in mS/cm^2^, resistivities in Ohm cm) of (584,100,000), (262,10,000), (584,10,000) and (262,100,000) respectively. As previously described, the simulation of myocardial volume effects was achieved by assuming that each one of the four ECGs could theoretically each represent one of three possible orthogonal ECGs within a hypothetical volume of myocardium. Vector addition of various combinations of 3 ECGs would therefore produce a resultant vector ECG incorporating the characteristics of the 3 constituent orthogonal waveforms. Four resultant ECGs were produced from the following combinations Res1 = ECG2+ECG3+ECG4, Res2 = ECG1+ECG2+ECG3, Res3 = ECG1+ECG3+(-ECG2), Res4 = ECG1+ECG2+(-ECG4). Res3 and Res4 simulated positive/negative biphasic ECGs. Figures 4, 5, 6 and 7 show the baseline ECGs Res1, Res2, Res3 and Res4 respectively without superadded noise. Vertical markers above the electric baseline show the ends of the T waves as calculated by each of the algorithms without any filtering. The T waves return to the isoelectric line at times 495, 461, 461 and 495 milliseconds from the onset of each QRS complex for ECGs Res1, Res2, Res3 and Res4 respectively. The real ends of the T waves are marked with vertical markers below the baselines. Table 1 shows the results of the calculated QT intervals by each of the algorithms without filtering, on each of the resultant ECGs (Res1, 2, 3 and 4) as shown in figures 4, 5, 6 and 7. Table 2 shows the results of the calculated QT intervals by each of the algorithms without filtering, on each of the resultant ECGs Res1, Res2, Res3 and Res4 downward shifted by 0.1 millivolts. It can be seen that threshold methods T1 and T2 are very sensitive to such a simulated shift in baseline, the slope algorithms S1 and S2 being less sensitive. The threshold method T3, which calculates the time at which the graph of the first differential of the T wave with respect to time crosses the zero baseline, is unaffected by shifting the baseline. Similarly the Novel algorithm is insensitive to manoeuvres such as baseline shift.

**Table 1:** Automatic algorithm calculation of QT intervals for unfiltered, non-amplified, non-noisy Resultant Vector ECGs. T1, T2, T3, S1, S2 and Novel are the algorithms as described in the text. Res1, Res2, Res3 and Res4 are the resultant vector ECG described in the text unfiltered, non-amplified and without added noise. The numerical values are in milliseconds.

	**Res1**	**Res2**	**Res3**	**Res4**
**T1**	445	404	353	345
**T2**	430	387	348	343
**T3**	496	462	371	386
**S1**	433	379	353	346
**S2**	435	382	355	347
**Novel**	495	461	461	495

**Table 2:** Automatic algorithm calculation of QT intervals for unfiltered, non-amplified, non-noisy Resultant Vector ECGs 0.1 millivolt downward shifted. T1, T2, T3, S1, S2 and Novel are the algorithms as described in the text. Res1, Res2, Res3 and Res4 are the resultant vector ECG described in the text unfiltered, non-amplified and without added noise with a 0.1 millivolt downward shift of the baseline. The numerical values are in milliseconds.

	**Res1**	**Res2**	**Res3**	**Res4**
**T1**	420	376	342	334
**T2**	415	365	339	332
**T3**	496	462	371	386
**S1**	420	366	343	335
**S2**	422	368	344	334
**Novel**	495	461	461	495

As previously discussed increases in conduction effects were simulated by a doubling of the amplitude at each time instant on the resultants ECGs Res1, Res2, Res3 and Res4 to give ECGs Res5, Res6, Res7 and Res8 respectively. The respective times at which the modelled T waves return to the isoelectric lines for these amplified ECGs is unchanged from the times given for their corresponding non-amplified resultant ECGs. Table 3 shows the results of the calculated QT intervals by each of the algorithms without filtering, on each of the amplified resultant ECGs Res5, Res6, Res7 and Res8. The calculated QT intervals by each of the algorithms, for each of these non-noisy, amplified, resultant ECGs, is exactly the same as the QT intervals calculated on the corresponding non-amplified resultant ECGs. All algorithms appear insensitive to uniform millivolt amplification of the T wave signal as would occur from pure conduction effects.

**Table 3:** Automatic algorithm calculation of QT intervals for unfiltered, amplified, non-noisy Resultant Vector ECGs. T1, T2, T3, S1, S2 and Novel are the algorithms as described in the text. Res1, Res2, Res3 and Res4 are the resultant vector ECG described in the text unfiltered, amplified by a factor of 2 and without added noise. The numerical values are in milliseconds.

	**Res5**	**Res6**	**Res7**	**Res8**
**T1**	445	404	353	345
**T2**	430	387	348	343
**T3**	496	462	371	386
**S1**	433	379	353	346
**S2**	435	382	355	347
**Novel**	495	461	461	495

**Figure 8:**
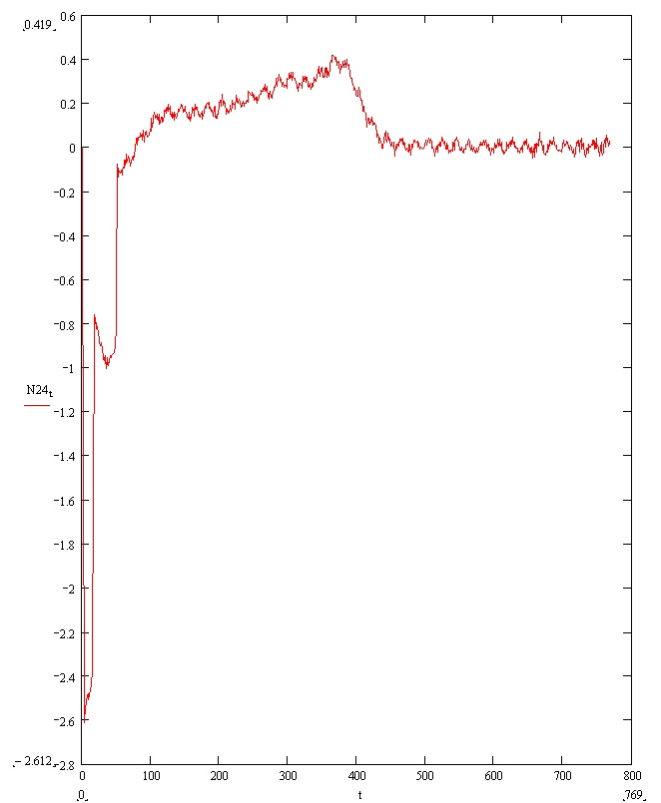
**Resultant Vector ECG Res1 with superadded noise**. The superadded noise consists of 30 dB SNR white noise, 30 dB SNR mains noise and respiratory rate of 30 per minute with 0.15 amplitude modulation and 3pi/2 phase. The y axis is in millivolts and the x axis is in milliseconds.

**Figure 9:**
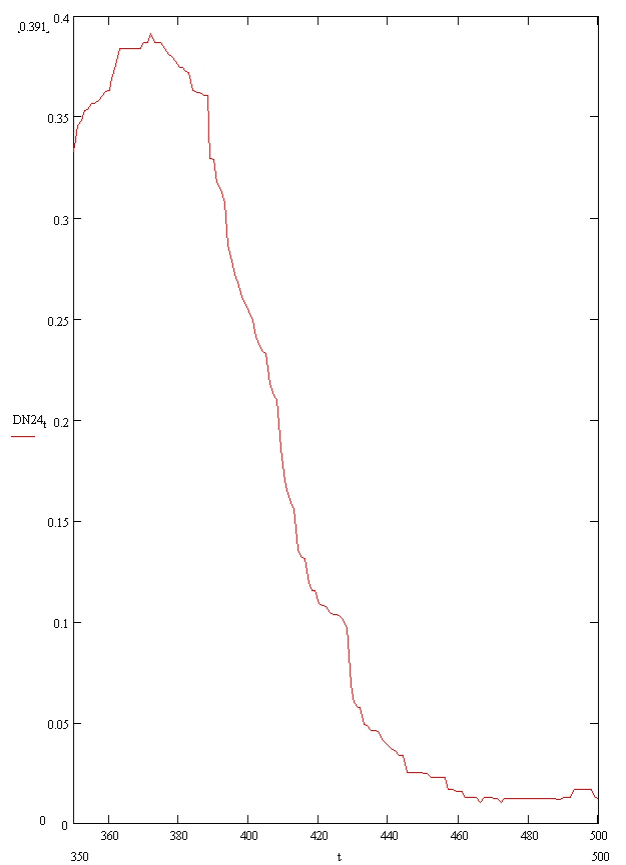
**Denoised T wave of Resultant Vector ECG Res1**. The magnified denoised T wave from Figure 10 is shown. The y axis is in millivolts and the x axis is in milliseconds.

Figure 8 shows resultant ECG Res1 with different combinations of superadded noise as described in the Legend. Figure 9 shows the magnified T wave from the filtered signal shown in Figure 8.

The means, (standard deviation) and %coefficients of variation for the measured QT intervals, on each of the baseline ECGs with superadded noise, using algorithms T1, T2, T3, SI, S2 and the Novel method are shown in Table 4. See Additional files 1, 2, 3, 4, 5, 6, 7 and 8 showing the results of QT intervals calculated by each of the algorithms on the noisy simulated ECGs; Res1, Res2, Res3, Res4, Res5, Res6, Res7 and Res8 respectively. The coefficients of variation were calculated as the percentage of the standard deviation divided by the mean value resulting from that particular algorithm method. The coefficients of variation for T1, T2, T3, S1, S2 and Novel were 0.36, 0.23, 1.9, 0.93, 0.92 and 0.62% respectively. All algorithms showed a high reproducibility and there was a significantly superior reproducibility shown by the algorithms T1, T2 and Novel compared to T3, S1 and S2. The two threshold methods T1 and T2 having a lower coefficient of variation than Novel.

**Table 4:** Calculation of mean QT intervals and coefficients of variation on all noisy Resultant Vector ECGs by all Automatic Algorithms. T1, T2, T3, S1, S2 and Novel are the algorithms as described in main body of text. Res1, Res2, Res3, Res4, Res5, Res6, Res7 and Res8 are the resultant vector ECGs as described in the main body of text. The mean values and their standard deviations are in milliseconds and the coefficients of variation are in percentages.

	**T1**	**T2**	**T3**	**S1**	**S2**	**Novel**
**Res1**						
**Mean**	448.72	431.74	466.39	426.04	446.69	493.62
**SD**	3.43	1.64	14.92	3.88	6.19	0.895
**%CV**	0.76	0.38	3.20	0.91	1.39	0.18
**Res2**						
**Mean**	406.67	388.46	429.92	365.63	401.16	462.28
**SD**	2.03	1.06	17.34	7.64	5.38	0.88
**%CV**	0.50	0.27	4.05	2.09	1.34	0.19
**Res3**						
**Mean**	352.59	348.85	370.44	350.61	364.09	462.87
**SD**	0.74	0.70	0.81	2.34	3.61	5.68
**%CV**	0.21	0.20	0.22	0.67	0.99	1.22
**Res4**						
**Mean**	346.10	343.44	385.33	346.83	348.42	490.10
**SD**	0.71	0.74	1.33	0.64	1.17	0.63
**%CV**	0.21	0.22	0.34	0.19	0.34	0.13
**Res5**						
**Mean**	447.03	430.49	462.51	427.14	443.69	495.41
**SD**	1.94	0.78	17.09	3.75	2.11	1.63
**%CV**	0.43	0.18	3.69	0.88	0.48	0.33
**Res6**						
**Mean**	406.05	388	433.85	367.32	399.15	469.54
**SD**	1.89	1.01	14.64	7.04	5.93	8.41
**%CV**	0.47	0.26	3.38	1.92	1.49	1.79
**Res7**						
**Mean**	352.15	348.74	370.77	351.88	361.12	463.72
**SD**	0.48	0.44	0.92	2.07	3.29	4.58
**%CV**	0.14	0.13	0.25	0.59	0.91	0.99
**Res8**						
**Mean**	345.64	343.03	385.21	346.59	348.2	493.15
**SD**	0.58	0.70	1.36	0.60	1.48	0.70
**%CV**	0.17	0.20	0.35	0.17	0.42	0.14

The four ECGs with real QT intervals of 495 milliseconds (Res1, Res4, Res5 and Res8) showed mean(standard deviations) QT intervals of 396.87(1.66), 387.175(0.965), 424.86(8.675), 386.65(2.218), 396.75(2.738) and 493(0.964) milliseconds when calculated by the respective algorithms T1, T2, T3, S1, S2 and Novel. The four ECGs with real QT intervals of 461 milliseconds (Res2, Res3, Res6 and Res7) showed mean(standard deviations) QT intervals of 379.37(1.285), 368.512(0.803), 401.25(8.43), 358.86(4.773), 381.53(4.553) and 464.6(4.888) milliseconds when calculated by the respective algorithms T1, T2, T3, S1, S2 and Novel. ANOVA showed that there was a significant difference between the accuracy of the QT algorithms at a >0.95 confidence level. The Tukey test showed that at the 0.95% confidence level the Novel method was significantly more accurate in measuring real QT interval than the other algorithms. The other algorithms demonstrated the following descendent order of accuracy when measuring ECGs with real QT intervals of 495 milliseconds: T3, T1, S2, T2 and S1. Similarly, the algorithms demonstrated the following descendent order of accuracy when measuring ECGs with real QT intervals of 461 milliseconds: T3, S2, T1, T2 and S1.

The effects of respiratory noise alone did not significantly affect the reproducibility of QT interval measurements made by any of the algorithms. Analysis of the contribution from either mains noise alone or white noise alone showed them to both be responsible for QT interval measurement instability.

## Discussion

This publication describes the first objective accurate physiological simulation reference standard employed to assess the accuracy of automatic computer algorithms to measure the QT interval. The reference method is based upon the synthesis of multiple ECG waveforms (derived from varying physiological parameters of resistivity and potassium channel conductance) by numerical solution of the one dimensional diffusion equation which describes current and voltage propagation through cardiac tissue [10]. The instantaneous cellular membrane currents and voltages being calculated by the thorough well founded simulations of the mammalian cardiac cell ventricular action potential derived from known ionic channel kinetics by Luo and Rudy [11]. The numerical method used to calculate the instantaneous membrane voltages was the Crank-Nicholson method which is known to be highly accurate and have good stability [16]. The four baseline ECGs (Figure 3) generated under conditions of modified potassium channel concentrations and resistivity produced T waves with a typical appearance. These four baseline ECGs were used to simulate orthogonal vector potentials at different angles to the direction of myocardial fibre orientation and through different layers of myocardial cell composition. These models are lumped models and therefore have the disadvantages of over generalisation, particularly if the investigator were interested in the electrocardiogram generated specifically from either the endocardial, M cells or epicardium. However this homogeneous approach is appropriate for the scope of the current paper and allows the description of different complexities in the myocardial repolarisation potentials generated from changes in specific and limited parameters which would arise from volume effects. Therefore it was hypothesised that each of the baseline ECGs could represent orthogonal vectors of myocardial potentials which when added in different combinations would give different resultant ECGs containing the characteristics of the three XYZ orthogonal components. The T waves generated from various hypothetical orthogonal ECGs combinations produced T wave signals of varying complexity as shown in figures 4, 5, 6 and 7. The positive/negative biphasic T waves shown in figures 6 and 7 are typical of the biphasic ECG repolarisation changes seen in-vivo. This vector resultant ECG will always contain the information of the longest QT interval from a 12 lead ECG recorded on the same myocardial volume. Indeed the information within a12 lead ECG taken on a given volume of myocardium is collapsed within the resultant ECGs from that same myocardium and is why 12 lead ECGs can be accurately reconstructed from three orthogonal vectors [21]. This is also why QT interval dispersion can be measured from orthogonal XYZ ECG vectors [22]. The scope of this study was to compare the accuracy and reproducibility of commonly used automatic QT measurement algorithms applied to eight different complexities of the scalar ECG T waves, using an accurate and entirely objective reference standard. From the above arguments, it can be appreciated that the analysis of the T waves from all 12 ECG leads was not integral to the aims of this research.

T wave inversion morphology has not been simulated, not because it poses any technical difficulty (it only requires making the baseline ECG arrays negative), but because it merely requires the squaring then square rooting of the arrays as part of pre-processing before subjecting the now upright T wave for analysis by the different algorithms. It would therefore not add any new complexity to the T waves subjected to analysis in this research.

The majority of algorithms in commercial ECG devices use a combination of leads of signals in an effort to increase signal to noise ratio as noise is a major reason for the inaccuracy and poor reproducibility of ECG interval measurement and is always present in varying degrees within ECGs taken in-vivo. Thus the use of noise simulation in order to assess the accuracy and reproducibility of ECG measurements obtained by automatic computer algorithms is commonly used [12,13,23]. This study has used established methods to produce superadded and signal amplitude modulated noise to contaminate the eight resultant ECG signals [12,13]. The types of noise and signal to noise ratios were chosen to be compatible with a resting ECG, the noise intensity levels for various physiological combinations of noise were chosen to cover a wide anticipated range without being excessive for resting conditions. Because only resting populations of ECGs were considered the study did not simulate noise artefact which would occur during ambulatory ECG recordings such as abrupt movement artefact nor was poor electrode contact noise simulated. However it is anticipated that the same Novel method could be used to accurately measure QT intervals on ambulatory ECGs with additional signal pre-processing techniques.

The commonly used algorithms which were compared in this research were based on those used in previous studies [14,20] and are those algorithms present in the majority of commercial ECG recording devices. Furthermore it is known that the slope method S2 is the most commonly used automatic algorithm in clinical ECG recorders. Previous methods used to assess different QT interval measuring algorithms have been either based on the reproducibility of the results obtained or upon the correlation with expert manual measurement which is analogous to measuring length with an elastic ruler. The methods used in assessing the algorithms cited in this report are objective, accurate and reproducible.

Table 1 shows the measured QT intervals by the various algorithms in the uncontaminated resultant ECG complexes. The Novel algorithm demonstrates a highly accurate QT measurement in both the monophasic and biphasic T wave complexes of Res3 and Res4. The T3 method is accurate for the monophasic Res1 and Res2 complexes but less accurate in the biphasic complexes. This occurs because the time of the negative phase plateau would be the time at which the first differential of the T wave crosses the isoelectric zero baseline, which is a time before the real end of the T wave. Similarly the negative phase of the T waves in Res3 and Res4 is responsible for the apparent shortening of the QT interval when calculated by T1, T2, S1 and S2, compared to measurements made by these algorithms on Res1 and Res2. Algorithms T1, T2, S1 and S2 generally underestimated the QT value and the Threshold method T1 shows the most accurate estimation out of these four methods.

Table 2 shows the measured QT intervals in the ECGs (Res1, 2, 3 and 4) with a depressed baseline. The Novel algorithm QT estimation is again highly accurate and unaffected by baseline depression. There continues to be the shortening effects of the calculated QT interval by all the algorithms for Res3 and Res4 versus Res1 and Res2 due to T wave biphasicity for all the algorithms. T3 remains unaffected and accurate for QT estimation in Res1 and Res2 because it is a threshold method which is dependent on the timing of the first differential with respect to time, which would not be affected by baseline depression.

The slope methods S1 and S2 are mildly affected by a depression in the ECG baseline because the point of maximum slope is also depressed and the unchanged projected slope gradient derived by both these methods will shorten the time of zero baseline intersection. The threshold methods T1 and T2 are most sensitive to a shift in baseline because small vertical movements in the low amplitude low frequency slope at the time of T wave offset will translate into large movements in time at which the T wave offset crosses the threshold. Table 3 demonstrates that amplification of the T wave secondary to conduction effects gives no significant changes in the results from the threshold methods. This is to be expected as the linear amplification of the T wave above the baseline has not altered the relative timings of the proportions of the T with respect to the non amplified signal. The slope methods are a function of amplitude and time and although the tangent at the point of maximum negative slope of the T wave increase with increasing T wave amplification, it increases directly in proportion to the increased amplitude of the T wave at the time of maximum down-slope i.e. the time from maximum down-slope to the time at which the tangents intersect the zero baseline is unchanged for the amplified and non amplified T waves.

The T3, S1 and S2 methods demonstrated the highest variability because they were all functions of a differential operator with respect to time on a smoothed signal with residual noise. It is known that any differentiation of a noisy signal lowers the signal to noise ratio thereby increasing the range of measured slope gradient, increasing the value of the maximum gradient and therefore increasing the potential for measurement error. The methods T1, T2 and Novel showed the least variability.

With the exception of the Novel method, the other five automatic algorithms demonstrated very poor accuracy. The method T3 showed the best accuracy out of the five algorithms because the method is in effect using the differential of the T wave signal to determine the minimum of the T wave, which in the absence of noise or any biphasic waveform would be significantly accurate

The Novel algorithm displayed a superior accuracy compared to the other measurement algorithms because the method uses the template of the T wave to in effect measure its own offset. It uses an inverted image of itself as a template to determine the first time at which the upright T wave and its inverted image return together to a common isoelectric baseline. This method therefore is the common measure of the physiological end for both the inverted and upright T waves, rather than using a surrogate marker for the end of the T wave as seen in the other algorithm methods. The Novel method is not dependent upon a preset amplitude threshold and is therefore not vulnerable to shifts in the ECG baseline like the threshold methods T1 and T2. The method does not depend upon differentiation and therefore is not vulnerable to errors arising from the biphasic waveform or the excessive noise produced by differentiation. The accuracy and reproducibility of the Novel method is susceptible to noise and although pre filtering does substantially reduce the effects of noise, recording of the ECG under optimally quiet conditions would further enhance its accuracy. As a result of the CSE study it has been emphasised that small variability rather than high accuracy is a desirable property of waveform recognition methods. It therefore follows that a combination of both these attributes is an even more desirable feature.

Although it is not in the scope of this present paper to closely examine and compare the expert manual clinical method of measuring the real simulated QT interval, it is apparent that a reader of this paper may not agree with the entirely objective, simulated T wave end point and may wish to shorten the T wave end point in order for it to be more reconciled with a conventional clinical QT interval estimation. This problem arises because the clinical measurer of the QT interval may not be visibly aware of the very low frequency low amplitude content of the terminal part of the T wave. The Novel algorithm can be used to satisfy this clinical concern by using an 85% value or negative adjusting constant of the Novel algorithm QT value. This therefore describes a QT interval fixed relatively to the QT interval as calculated by the Novel algorithm. This can be appreciated in Figure 2, which shows the 85% Novel algorithm QT in both the raw clinical ECG signal and in the post filtered ECG T wave. This topic will be the subject of future research as it is not in the scope of the present paper.

## Conclusion

A new validated reference standard has been formulated based on sound physiological principles which enabled the objective assessment of the accuracy and reproducibility of commonly used automatic computer algorithms which measure the scalar ECG QT interval. A new Novel computer algorithm has also been introduced.

The commonly used computer algorithms were all shown to be inaccurate in measuring the real QT interval. The Novel algorithm demonstrated accuracy in measuring the QT interval with high reproducibility. Two threshold methods were shown to give highly reproducible but inaccurate QT interval measurements which were prone to large variable errors with ECG baseline fluctuation.

## Competing interests

This paper was written with the pure scientific intention of assessing the accuracy and reproducibility of commonly used automatic computer algorithms which measure the QT interval using a new objective, reproducible and accurate reference standard. A new more accurate algorithm has been introduced which may lead to interest and further developmental research being performed. The Novel algorithm does not have a registered patent. In the long term the published results of this research may reflect favourably on PSI Heartsignals Ltd which may in turn lead to financial benefit but that could be said of any research.

## Authors' contributions

The author of this paper was entirely responsible for the initial concept, the writing of the computer software and final preparation of the manuscript.

## Pre-publication history

The pre-publication history for this paper can be accessed here:



## Supplementary Material

Additional File 1 **Automatic algorithm calculation of QT intervals for Resultant Vector ECG Res1 with superadded noise **T1, T2, T3, S1, S2 and Novel are the algorithms as described in the main body of text. N1 to N39 are 39 different combinations of noise as described in the main body of the text superadded to ECG Res1. The numerical values are in milliseconds. The real QT interval is 495 milliseconds. Click here for file

Additional File 2 **Automatic algorithm calculation of QT intervals for Resultant Vector ECG Res2 with superadded noise **T1, T2, T3, S1, S2 and Novel are the algorithms as described in the main body of text. N1 to N39 are 39 different combinations of noise as described in the main body of the text superadded to ECG Res2. The numerical values are in milliseconds. The real QT interval is 461 milliseconds. Click here for file

Additional File 3 **Automatic algorithm calculation of QT intervals for Resultant Vector ECG Res3 with superadded noise **T1, T2, T3, S1, S2 and Novel are the algorithms as described in the main body of text. N1 to N39 are 39 different combinations of noise as described in the main body of the text superadded to ECG Res3. The numerical values are in milliseconds. The real QT interval is 461 milliseconds. Click here for file

Additional File 4 **Automatic algorithm calculation of QT intervals for Resultant Vector ECG Res4 with superadded noise **T1, T2, T3, S1, S2 and Novel are the algorithms as described in the main body of text. N1 to N39 are 39 different combinations of noise as described in the main body of the text superadded to ECG Res4. The numerical values are in milliseconds. The real QT interval is 495 milliseconds. Click here for file

Additional File 5 **Automatic algorithm calculation of QT intervals for Resultant Vector ECG Res5 with superadded noise **T1, T2, T3, S1, S2 and Novel are the algorithms as described in the main body of text. N1 to N39 are 39 different combinations of noise as described in the main body of the text superadded to ECG Res5. The numerical values are in milliseconds. The real QT interval is 495 milliseconds. Click here for file

Additional File 6 **Automatic algorithm calculation of QT intervals for Resultant Vector ECG Res6 with superadded noise **T1, T2, T3, S1, S2 and Novel are the algorithms as described in the main body of text. N1 to N39 are 39 different combinations of noise as described in the main body of the text superadded to ECG Res6. The numerical values are in milliseconds. The real QT interval is 461 milliseconds. Click here for file

Additional File 7 **Automatic algorithm calculation of QT intervals for Resultant Vector ECG Res7 with superadded noise **T1, T2, T3, S1, S2 and Novel are the algorithms as described in the main body of text. N1 to N39 are 39 different combinations of noise as described in the main body of the text superadded to ECG Res7. The numerical values are in milliseconds. The real QT interval is 461 milliseconds. Click here for file

Additional File 8 **Automatic algorithm calculation of QT intervals for Resultant Vector ECG Res8 with superadded noise **T1, T2, T3, S1, S2 and Novel are the algorithms as described in the main body of text. N1 to N39 are 39 different combinations of noise as described in the main body of the text superadded to ECG Res8. The numerical values are in milliseconds. The real QT interval is 495 milliseconds. Click here for file
