# Supplementary material for: Accuracy of popular automatic QT Interval algorithms assessed by a 'Gold Standard' and comparison with a Novel method: computer simulation study
Source: BMC Cardiovasc Disord. 2005 Sep 26;5:29. doi: 10.1186/1471-2261-5-29 (PMC1262700; doi:10.1186/1471-2261-5-29)
Supplement: Additional File 4 — Automatic algorithm calculation of QT intervals for Resultant Vector ECG Res4 with superadded noise T1, T2, T3, S1, S2 and Novel are the algorithms as described in the main body of text. N1 to N39 are 39 different combinations of noise as described in the main body of the text superadded to ECG Res4. The numerical values are in milliseconds. The real QT interval is 495 milliseconds. [file 1471-2261-5-29-S4.doc]

|  | **T1** | **T2** | **T3** | **S1** | **S2** | **Novel** |
| --- | --- | --- | --- | --- | --- | --- |
| **N1** | 345 | 343 | 384 | 346 | 347 | 490 |
| **N2** | 346 | 343 | 385 | 346 | 347 | 490 |
| **N3** | 346 | 343 | 387 | 347 | 348 | 491 |
| **N4** | 346 | 343 | 384 | 347 | 350 | 490 |
| **N5** | 346 | 343 | 384 | 347 | 349 | 490 |
| **N6** | 346 | 343 | 384 | 347 | 350 | 490 |
| **N7** | 345 | 343 | 384 | 346 | 348 | 491 |
| **N8** | 345 | 343 | 384 | 346 | 348 | 490 |
| **N9** | 345 | 343 | 384 | 346 | 348 | 489 |
| **N10** | 345 | 343 | 384 | 346 | 348 | 490 |
| **N11** | 345 | 343 | 384 | 346 | 348 | 489 |
| **N12** | 345 | 343 | 384 | 346 | 348 | 490 |
| **N13** | 347 | 344 | 387 | 347 | 348 | 490 |
| **N14** | 347 | 344 | 387 | 347 | 348 | 490 |
| **N15** | 347 | 345 | 385 | 347 | 349 | 490 |
| **N16** | 346 | 342 | 386 | 345 | 354 | 490 |
| **N17** | 346 | 343 | 387 | 347 | 347 | 491 |
| **N18** | 347 | 343 | 385 | 347 | 349 | 490 |
| **N19** | 346 | 343 | 383 | 348 | 347 | 489 |
| **N20** | 346 | 344 | 387 | 347 | 348 | 490 |
| **N21** | 347 | 345 | 386 | 347 | 349 | 489 |
| **N22** | 346 | 343 | 383 | 348 | 347 | 490 |
| **N23** | 346 | 344 | 387 | 347 | 348 | 490 |
| **N24** | 348 | 344 | 387 | 346 | 348 | 490 |
| **N25** | 346 | 343 | 384 | 347 | 350 | 490 |
| **N26** | 346 | 343 | 387 | 347 | 347 | 491 |
| **N27** | 347 | 345 | 385 | 347 | 349 | 491 |
| **N28** | 346 | 343 | 384 | 347 | 349 | 490 |
| **N29** | 346 | 343 | 387 | 347 | 347 | 491 |
| **N30** | 347 | 343 | 385 | 347 | 349 | 491 |
| **N31** | 346 | 343 | 384 | 347 | 348 | 489 |
| **N32** | 346 | 344 | 387 | 347 | 348 | 490 |
| **N33** | 347 | 345 | 386 | 347 | 349 | 490 |
| **N34** | 346 | 343 | 384 | 348 | 347 | 489 |
| **N35** | 346 | 344 | 387 | 347 | 348 | 490 |
| **N36** | 347 | 345 | 386 | 347 | 349 | 490 |
| **N37** | 346 | 343 | 384 | 347 | 349 | 491 |
| **N38** | 346 | 343 | 387 | 347 | 347 | 491 |
| **N39** | 346 | 344 | 387 | 347 | 348 | 491 |
